# Integration of Electrical Signals and Phytohormones in the Control of Systemic Response

**DOI:** 10.3390/ijms24010847

**Published:** 2023-01-03

**Authors:** Maria Ladeynova, Darya Kuznetsova, Maxim Mudrilov, Vladimir Vodeneev

**Affiliations:** Department of Biophysics, National Research Lobachevsky State University of Nizhny Novgorod, 23 Gagarin Avenue, 603950 Nizhny Novgorod, Russia

**Keywords:** long-distance signaling, electrical signals, hormones, jasmonate, calcium, systemic response, plants

## Abstract

Plants are constantly exposed to environmental stresses. Local stimuli sensed by one part of a plant are translated into long-distance signals that can influence the activities in distant tissues. Changes in levels of phytohormones in distant parts of the plant occur in response to various local stimuli. The regulation of hormone levels can be mediated by long-distance electrical signals, which are also induced by local stimulation. We consider the crosstalk between electrical signals and phytohormones and identify interaction points, as well as provide insights into the integration nodes that involve changes in pH, Ca^2+^ and ROS levels. This review also provides an overview of our current knowledge of how electrical signals and hormones work together to induce a systemic response.

## 1. Introduction

Plants are in continuously changing environmental conditions and are exposed to a wide range of stresses, caused by exposure to extremes of temperature, excessive light, limited water and nutrient availability, pathogens and herbivore attacks. Environmental stimuli can arrive in a spatially and temporally heterogeneous manner, and so are sensed in separate plant parts, which perceive and transmit information about the stimulus to distant parts to induce a systemic adaptive response. Information about the stimulus is translated into a long-distance mobile signal, which influences the activity of processes in distant tissues. Plants can coordinate developmental or defense processes throughout their bodies, using hydraulic, electrical and chemical long-distance signals, which differ in their nature and speed of propagation [1].

A hydraulic signal is a wave of increased hydraulic pressure, which rapidly propagates through xylem vessels [1,2]. Pressure changes in xylem vessels are perceived by adjacent parenchyma cells as a mechanical signal, mediated by turgor pressure changes in these cells and by changes in membrane tension [3]. The propagation speed of hydraulic signals is high and can reach the speed of sound in water for a pressure wave, and tens of cm/s for hydraulic mass flow in xylem [1,2]. Electrical signals (ESs) are transient changes in the membrane potential, which quite rapidly propagate through tissues of the plant. The type of electrical signal depends on the type of the stimulus and ion fluxes involved in its generation. There are action potential (AP), variation potential (VP) and system potential (SP). Undamaging local stimuli (cold, touch, light/shadow) induce AP, short-term depolarization with a spike shape. Damaging local stimuli (wounding, burning, heating) induce VP, long-term transient depolarization which has an irregular shape. SP is a transient hyperpolarization generated in response to various stimuli. The propagation speed of ESs in plants ranges from a few mm to several cm per second and depends on the type of signal and plant species [1,2,4,5,6,7,8]. Chemical signals are transient changes in the concentration of various compounds, which include propagating waves of reactive oxygen species (ROS), changes in ion concentrations, such as Ca^2+^ waves, and also phytohormones, peptides and various volatile compounds. Chemical signals propagate through the plant at low speed, in general, from a few µm/s to several hundreds or thousands of µm/s [1,2,7].

It is considered that information about environmental stressors is not transmitted by a single pathway, but rather by a combination of signals, which can transmit information about the nature of the stressor and its intensity [1,7,9]. Crosstalk between hydraulic, electrical and chemical signaling pathways is critical in information processing for the induction of the systemic response. In this review, we focus on electrical and hormone signaling, their interplay and their integration in the systemic response to local stressors.

The change in electrical activity is a universal and fast plant reaction to external stimuli. The rapid propagation of ESs at a long distance is critical to a plant organism’s ability to coordinate a wide range of physiological processes, including the production of phytohormones [7,10], which are essential regulators of growth, development and defense responses. The change in the phytohormone concentration leads to a specific physiological response, which may be mediated by changes in the electrical activity, caused, in turn, by the activation of hormone signaling pathways. The interplay between ESs and phytohormones is increasingly confirmed in the literature; evidence for this concept is given in this review.

**Figure 1:**
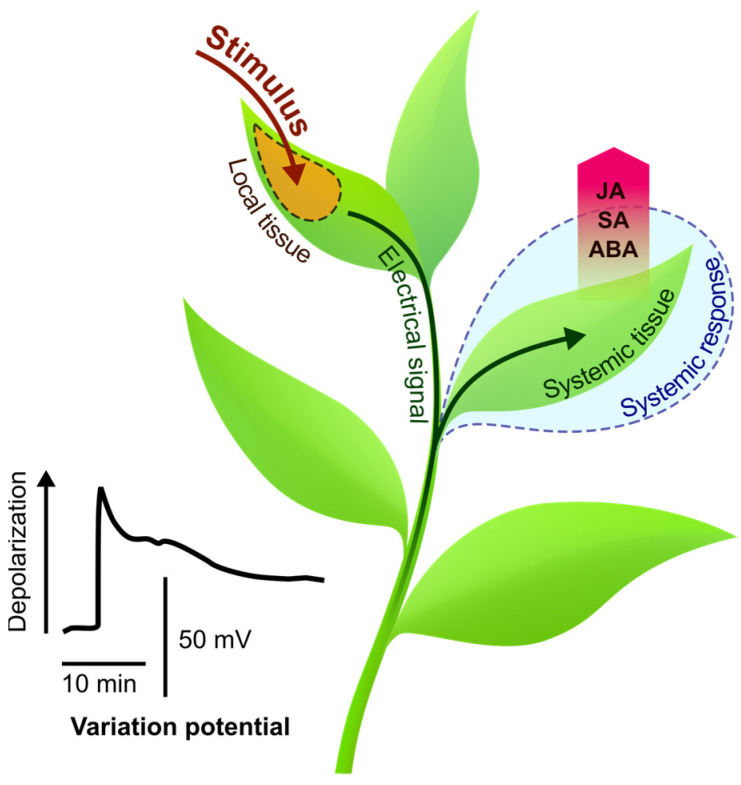
Long-distance electrical signal (e.g., variation potential) occurs in response to a local stimulus and propagates from the local tissue to the systemic tissue, where it controls stimulus-induced changes in hormone levels, and then the electrical signal and hormones together regulate stimulus-induced systemic response. ABA, abscisic acid; JA, jasmonic acid; SA, salicylic acid.

Here, we discuss the regulation of ES-induced changes in the phytohormone levels, and we begin with a review of the spatiotemporal dynamics of phytohormones in response to local stimuli, as the characteristic temporal and spatial patterns of changes in hormone levels provide information about potential mechanisms that mediate these changes. Finally, we present specific molecular mechanisms regulating changes in the phytohormone levels by ESs, which demonstrate direct evidence of crosstalk and provide a mechanistic understanding of the link between hormonal and electrical signaling systems. We also discuss integration ways of electrical and hormone signals in the regulation of systemic response at its different phases. The integration of signaling events, mediated by interplay between ESs and phytohormones, results in the formation of systemic response (Figure 1) and, finally, in increased resistance to stress.

## 2. Electrical Signals Are Involved in the Regulation of Stimulus-Induced Changes in Phytohormone Levels

### 2.1. Spatiotemporal Dynamics of Phytohormones in Response to Local Stimuli

Various local stresses induce changes in the content of phytohormones. Temporal and spatial dynamics are individual characteristics of each hormone that represent their particular physiological function and potential contribution to the systemic response. Understanding the spatiotemporal dynamics of phytohormones is necessary both to elucidate the role of individual hormones in the initiation and regulation of the physiological response and to determine potential ways of regulating phytohormone production. In this section, we summarize studies of the spatiotemporal dynamics of stress phytohormones in response to various local stimuli in the stimulation zone and outside this zone. Here, we focus mainly on the dynamics of jasmonates (JAs), abscisic acid (ABA) and salicylic acid (SA) as key plant hormones in response to local abiotic and biotic stresses [11,12,13].

Local stimuli induce changes in the phytohormone content both in tissues which are directly stressed in the stimulation zone, termed ‘local tissues’, and in tissues located at a long distance from this zone, termed ‘systemic tissues’. It is known that local and systemic changes in the concentration of phytohormones occur under the actions of local stimuli such as wounding [14,15,16,17,18,19,20,21,22,23,24,25,26,27,28,29,30,31], light stress (high light) [32,33], heat stress [33,34,35], burning [14,35,36,37,38,39] and herbivore attack or mechanical wounding with insect oral secretion treatment [15,25,26,30,40,41,42,43]. The time interval of phytohormone quantification after a local stimulation varies widely, from tens of seconds or a few minutes to several hours or days.

**Figure 2:**
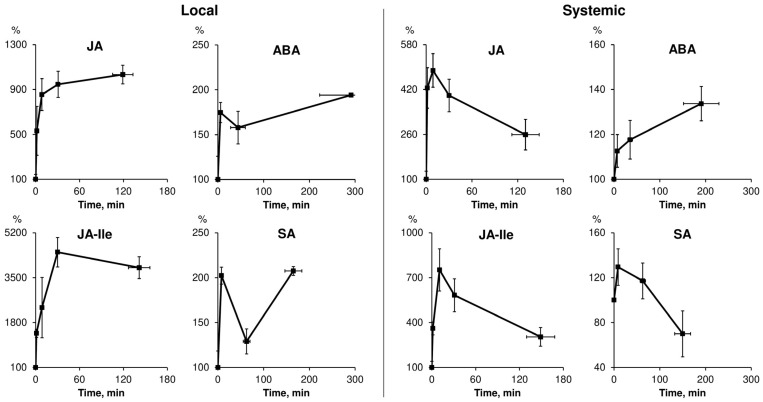
Dynamics of levels of jasmonic acid (JA), jasmonoyl-L-isoleucine (JA-Ile), abscisic acid (ABA) and salicylic acid (SA), induced by a local stimulus, in local and systemic plant tissues; the moment of stimulation is the 0 min time point. Hormone levels are presented as % of the resting level (0 min) and are calculated by averaging the amplitudes of changes in hormone levels. Time points are calculated by averaging time points. Values are means ± SEM of 3–20 independent studies. References for all studies included in this analysis can be found in the text of Section 2.1. Local: JA [14,15,16,17,18,20,21,22,25,26,27,28,30,32,33,37,41], JA-Ile [15,16,18,19,21,25,26,27,28,30,40], ABA [14,17,28,32,37], SA [15,17,20,25,26,28,32,33]. Systemic: JA [18,19,21,23,24,27,29,32,33,35,36,39], JA-Ile [18,19,21,23,24,27,28,29,40], ABA [14,28,32,34,35,36,37,39], SA [28,32,34,35,36].

The most studied phytohormones, known for their involvement in responses to local stimuli, are JAs. The content of JAs rapidly increases after stimulation in local and systemic tissues (Figure 2). It is shown that the levels of jasmonic acid (JA) and its biologically active derivative, jasmonoyl-L-isoleucine (JA-Ile), increase within 0.7–5 min [16,19,22,27,29,32,33,35]. JA and JA-Ile attain peak levels generally in the time interval from 10 to 60 min in both local and systemic tissues [15,17,18,19,20,22,23,24,25,26,28,29,32,35,36,39,40,41]. After the peak, there is a gradual decline in the JA and JA-Ile content to resting levels, which is well expressed in systemic tissues, and is not always observed in local tissues (Figure 2). In some works, there is information about a later reaching of the peak levels of JA and JA-Ile in local tissues at 90 min after wounding [15,16,30], mechanical wounding with insect oral secretion treatment or herbivore attack [15,30,40]. On the other hand, the concentration of JA-Ile in some cases can remain high in local tissues for a long time after wounding; JA-Ile attains peak levels 360 min after stimulation [16,21].

The temporal dynamics of JA concentration in local and systemic tissues is generally similar, but quantitative differences in the levels of JAs are well expressed, which can be shown from works, where local and systemic changes in the concentration of JAs after the stimulation are studied simultaneously. In general, quantitatively, local changes exceed systemic ones (Figure 2). This dependence is characteristic of different types of local stimuli: mechanical wounding [14,18,19,21,27,28,30], light stress [32], burning [37] and the combined application of mechanical wounding and insect oral secretion [15,30]. However, there are single oppositive examples, which demonstrate that the local stimulus can induce a more expressed increase in JA concentration in systemic tissues than in local tissues. This dynamics is observed under high light treatment in Arabidopsis: JA levels reach a maximum 2 min after stimulation, and peak levels of JA in the systemic tissue were ~twofold higher than in the local tissue [33].

The dynamics of ABA has been studied in a much smaller number of works compared to JAs, and available information is rather contradictory. A local stimulus, generally, causes an increase in the content of ABA in local and systemic tissues (Figure 2) [14,17,31,32,34,35,36,37,39]. There is information about the significant decrease in ABA concentration in the systemic leaf of *Arabidopsis* at 60 min after mechanical wounding compared with resting levels [28]. Meanwhile, ABA levels in systemic leaves may not change after local burning in tomato plants [37]. The ABA content in local and systemic tissues reaches its maximum in the time interval from 60 to 360 min [14,17,35,37,39]. However, there is evidence of a rapid (up to 15 min) increase in ABA concentration in local and systemic tissues upon high light treatment [32] and in systemic tissues upon burning [36]. It is difficult to determine exactly quantitative differences in the local and systemic dynamics of ABA; single works show that the increase in ABA concentration after stimulation in local tissues is more pronounced than in systemic tissues upon burning [37] and wounding [14].

The available studies on the dynamics of SA in response to local stimulation are rather few and contradictory. It is difficult to conclude about the features of the spatial and temporal dynamics of SA based on the limited data in the literature. Nevertheless, generally, SA concentration increases in local and systemic tissues in response to local stimuli (Figure 2) [15,17,25,26,32,33,35]. In some studies, burning [36] and wounding [28,31] have no effect on SA levels in local and systemic tissues. SA concentration can reach its maximum in a relatively short time interval up to 40 min in systemic tissues [35]. In local tissues, the maximum concentration of SA is generally in the time interval from 60 to 180 min [15,20,25,26]. A quantitative comparison of local and systemic changes shows that in response to high light or heat treatment, the increase in SA concentration 8 min after stimulation in systemic *Arabidopsis* leaves has a greater amplitude compared to changes in local leaves [33]. In another work [32], the increase in SA content at 10 min in response to high light had comparable amplitudes in local and systemic leaves in *Arabidopsis*.

Studies of the dynamics of other phytohormones, with the exception of JA, JA-Ile, ABA and SA, in response to local stimuli are presented by single works. Wounding of *Nicotiana tabacum* leaves causes a decline in levels of endogenous indole-3-acetic acid (IAA) in wounded and unwounded regions of the leaf at 180 and 360 min time points [44]. Wounding of *Arabidopsis thaliana* leaves did not cause local changes in IAA content at 30 min [31], 60 min and 360 min [17] after stimulation. The levels of gibberellins in local leaves of *Arabidopsis* were also unchanged at 60 min and 360 min after mechanical wounding [17].

It is assumed that 12-oxo-phytodienoic acid (OPDA) is not only a JA precursor, but also a signaling molecule that activates OPDA-specific responses in an JA-independent manner [45]. In general, the local stimulus causes the increase in OPDA content in a wide time interval from 10 to 360 min in local and systemic tissues [17,22,25,30,41]. There is information about the rapid (<5 min) increase in OPDA levels in local and systemic tissues in response to high light [32], wounding [22] and after simulated herbivory treatments [41]. Comparison of local and systemic OPDA dynamics induced by a local wounding shows that in damaged local leaves, there is a gradual increase in OPDA concentration from 5 to 60 min after wounding, whereas in undamaged systemic leaves, OPDA levels rapidly decrease within 5 min and then slowly increase until 180 min [19]. In another work, OPDA levels were increased only in the local leaf 60 min after mechanical wounding [28]. There is also information that wounding does not affect the OPDA content in the local leaf 30 min after stimulation [31].

Thus, it is clearly seen that along with a sufficiently detailed study of the dynamics of JAs in response to local stimuli, works on the dynamics of ABA and SA are insufficient, and studies of the dynamics of other hormones are in its infancy and need further study. In particular, studies of the spatial dynamics of hormones in wide time intervals using different plant species are needed to confirm the universality/specificity of the observed patterns.

#### 2.1.1. Specificity of Phytohormone Dynamics Induced by Local Stimuli of Different Nature

Phytohormone dynamics in local and systemic tissues depend on the nature of the local stimulus (Box 1), which can be demonstrated by a number of examples. Correct comparison of changes in phytohormone content in response to different local stimuli is possible only when measurements are performed within the same work on the same plant material under similar conditions. Wounding causes a slightly greater increase in ABA concentration compared with burning in local tomato leaves, whereas JA levels increase equally in response to burning and wounding [14]. In *Arabidopsis*, heat stress induces a more pronounced increase in the JA content in local leaves compared to high light treatment [33]. In systemic leaves, the reverse is observed: only light stress causes an increase in JA levels, whereas heat stress does not cause changes in JA concentrations [33]. For SA, there is no specificity of dynamics in both local and systemic leaves [33]. Local gradual heating induced a more pronounced increase in JA, ABA and SA levels compared with local burning in systemic tissues of wheat plants; the temporal dynamics between stimuli were similar [35].

Box 1 **Stimuli that induce phytohormone production.**

JA

ABA

SA

**Herbivore attack**

**Drought**

**Pathogen attack**

**Pathogen attack**

**Heat stress**
Light stress
**Mechanical wounding**
Cold stressHeat stressLight stressLight stressHerbivore attackHeat stressMechanical woundingSalt stressDroughtPathogen attackCold stressCold stressSalt stressDroughtOzone stressEtc.Etc.Heavy metal stress

Salt stress

Etc.

The specificity of hormone dynamics is best studied upon herbivore attack, the effects of which are complex and include mechanical wounding and elicitors of insect oral secretion. In local leaves of *Arabidopsis*, simulating an attack by the herbivorous insect *Spodoptera littoralis* causes a greater increase in JA and JA-Ile concentrations compared with mechanical wounding [25,30,46]; there are no such differences in the SA content [25]. Simulating an attack by the herbivorous insect *Manduca sexta* also induces a more pronounced increase in the JA and JA-Ile content [15,26], and in SA content [15] in local leaves of tobacco plants.

Differences in the dynamics of stress hormones, especially those expressed in quantitative changes, under the actions of stimuli of different nature indicate the role of phytohormones in the transmission of stimulus-specific information; therefore, hormones may be one of the necessary mediators in the regulation of the stimulus-specific systemic response. In turn, the specificity of phytohormone dynamics is most likely due to the specificity of long-distance signals, in particular ESs [9], which propagate from the stimulation site and induce changes in the phytohormone content in unstimulated systemic tissues.

### 2.2. Mechanisms of Regulation of Changes in Phytohormone Levels by Electrical Signals

The stimulus-induced systemic accumulation of phytohormones could result from the transport of phytohormones from damaged to undamaged parts of the plant or from the production of phytohormones directly in systemic tissues from precursors or conjugated forms (Box 2), in response to a long-distance signal. Phytohormones are capable of long-distance transport [47], but they have propagation speeds that are slow in comparison with hydraulic and ESs [1,2,7], suggesting that hormones are not long-distance communicators in a rapid systemic response. The data on the rapid accumulation of some phytohormones in systemic tissues in response to local stimuli discussed in the previous section, taking into account the low speed of phytohormone transport, rather indicate the production of hormones directly in systemic tissues. Among the phytohormones, JAs display the fastest (within a few minutes) accumulation in the distal parts of the plant in response to local stimuli. Therefore, the stimulus-induced systemic accumulation of phytohormones is the result of de novo synthesis of these phytohormones in systemic tissues, and not of the transport from the stimulation zone. This suggests the existence of a rapid long-distance signal that is generated in response to the stimulus, propagates from the site of stimulation to distal tissues, and activates hormone production in these tissues.

Box 2  

JA biosynthesis and metabolism

All steps in the biosynthesis of jasmonates (JAs) take place in three different compartments: the chloroplast, peroxisome and cytosol. The first stage occurs in the chloroplast, where a precursor, α-linolenic acid (18:3), is released from lipids of plastid membranes by hydrolases. Linolenic acid is then converted to 12-oxo-phytodienoic acid (OPDA) through several intermediate stages such as oxygenation by a lipoxygenase, transforming by allene oxide synthase, and cyclizing by allene oxide cyclase. OPDA is transported into the peroxisome, where it is reduced by 12-oxo-phytodienoic acid reductase, and then jasmonic acid (JA) is synthesized through three series cycles of β-oxidation. The last step takes place in the cytosol, where JA is able to form many different derivatives which are collectively called JAs. One of the most well-studied biological active derivatives of JA is a conjugate of JA with isoleucine, jasmonoyl-L-isoleucine (JA-Ile). Another derivative of JA, methyl ester (MeJA), is a volatile compound and has the ability to easily penetrate the membrane and airborne transmission to distal leaves or to other plants. JA and JA-Ile can be inactivated into many derivatives.

ABA biosynthesis and metabolism

Abscisic acid (ABA) is synthesized in plants through a carotenoid pathway. All stages of ABA biosynthesis require two reaction sites, plastids and cytosol. In plastids, the process of converting the precursor, β-carotene, into xanthoxin occurs through the formation of intermediate compounds, namely, zeaxanthin, violoxanthin and neoxanthin. In the cytosol, xanthoxin transforms into abscisic aldehyde by alcohol dehydrogenase, in the next step abscisic aldehyde oxidase converts abscisic aldehyde to ABA. It is known that ABA can be converted to an inactivated state, ABA-glucose ester (ABA-GE), by glycosylation in cytosol. In addition, a rapid reverse process to release active ABA by β-glucosidases from ABA-GE in the endoplasmic reticulum and vacuole is possible under the action of environmental factors.

SA biosynthesis and metabolism

Plants synthesize salicylic acid (SA) using two different pathways, the isochorismate synthase (ICS) pathway and phenylalanine ammonia-lyase (PAL) pathway. The precursor of SA in each of the synthesis pathways is chorismate. In the SA synthesis via the ICS pathway, on the first step chorismate is converted to isochorismate by ICS in the plastids. Then, isochorismate is transported to the cytosol and converted to SA through intermediate compounds such as isochorismic acid and isochorismate-9-glutamate. In the PAL pathway, phenylalanine transforms to cinnamate by PAL, then cinnamate can be converted to SA through benzoate. Like other phytohormones, SA can metabolite to active and inactive derivatives; inactive forms, SA glucoside and SA glucose ester, storage in a vacuole and can be hydrolyzed to active forms.

ESs that propagate from the stimulation site to the distal parts of the plant are well suited for the role of rapid long-distance signals that induce the systemic change in the content of hormones under local stimuli [10,19,48]. The regulation of hormone production may be mediated by changes in Ca^2+^ and ROS levels and by pH shifts, which accompany the generation and the propagation of ESs [4,7,49]. In this section, we consider potential ways of regulating changes in the phytohormone concentration by ESs, focusing mainly on the interconnected Ca^2+^ and ROS waves and pH shifts as key elements of this regulation.

Intensive research over the past two decades has identified important molecular components that may regulate JA metabolism under local stress. The largest amount of information was obtained in Ca^2+^-dependent regulation of JA metabolism. Changes in cytosolic Ca^2+^ concentration ([Ca^2+^]_cyt_) are provided by Ca^2+^ channels. Pretreating of the wounded leaf with the Ca^2+^ channel inhibitor La^3+^ blocked both the systemic [Ca^2+^]_cyt_ increase and systemic induction of wound-related marker genes *JASMONATE ZIM-domain (JAZ) 5, JAZ7* and *12-oxo-phytodienoic acid reductase (OPR) 3* [29], which are the primary response genes in the JA signaling pathway [19]. JA biosynthesis induced by heat-stimulated AP was suppressed by pretreatment with the Ca^2+^ channel blocker ruthenium red, which also suppressed the transient [Ca^2+^]_cyt_ elevation that was associated with AP [50].

Specific Ca^2+^ channels involved in the regulation of production of JAs have only just begun to be defined. Ca^2+^ channel CYCLIC NUCLEOTIDE GATED CHANNEL 19 (CNGC19) is rapidly activated upon wounding, resulting in the increase in [Ca^2+^]_cyt_. CNGC19 is involved in the regulation of JA biosynthesis upon herbivory, as demonstrated by studies with mutants, in which loss of *CNGC19* function resulted in both the decrease in stimulus-induced [Ca^2+^]_cyt_ elevation and the decrease in accumulation of JA and JA-Ile [43]. Similar results on the role of CNGC19 were also obtained when exposed to the stimulus of a different nature, colonization by *Piriformospora indica*, a root endosymbiont [51], which indicates the universal role of CNGC19 in the regulation of JA production under various stimuli. In addition, this work shows that the loss of *CNGC19* function affects not only the decrease in stimulus-induced JA and JA-Ile levels, but also the decrease in levels of their precursor, OPDA [51].

Another source of [Ca^2+^]_cyt_ elevations in a plant cell and the potential regulator of JA production is annexins, plasma membrane-localized proteins, which can exhibit Ca^2+^ channel-like activity. ANNEXIN 1 (ANN1) is involved in the [Ca^2+^]_cyt_ elevation in systemic leaves in response to local wounding, and also regulates the systemic increase in concentration of JAs. In *ann1* loss-of-function plants, the stimulus-induced increase in [Ca^2+^]_cyt_ was suppressed, and JA, JA-Ile and OPDA levels, increased in response to wounding, were significantly lower than in wild-type plants [30].

Cation channels of the GLUTAMATE RECEPTOR-LIKE (GLR) family are the important element in the generation and the propagation of VP and are involved in the [Ca^2+^]_cyt_ elevations under the local stimulation [7]. GLR3.5-dependent electrical signaling is essential for JA biosynthesis upon root-knot nematode attack in tomato plants [52]. It was also shown that GLRs affect parameters of wound-induced VP and control the systemic wound-induced expression of several key JA-inducible regulators of JA signaling (JAZ genes) in arabidopsis plants [48]. GLR3.3 positively regulates the wound-induced accumulation of JA and JA-Ile in systemic tissues in arabidopsis plants, but is not involved in the production of JAs in local tissues [53], which is consistent with the role of GLR3.3 in the VP propagation to systemic tissues [29,48]. GLR1.2 and GLR1.3 are positive regulators of JA biosynthesis upon cold stress [54]. As seen, GLRs mediating [Ca^2+^]_cyt_ elevation are universal positive regulators of JA production under various biotic and abiotic stresses, although one study also showed that GLR-mediated Ca^2+^ waves alone are insufficient to trigger JA signaling [55].

Another potential regulator of JA production that mediates [Ca^2+^]_cyt_ elevations in a plant cell and is involved in systemic propagation of the Ca^2+^ signal is tonoplast-localized TWO-PORE CHANNEL 1 (TPC1) [7,56]. *Arabidopsis* gain-of-function *fou2* (*fatty acid oxygenation upregulated 2*) mutant has the hyperactive version of TPC1 and increased JA and OPDA levels in resting leaves, which increase even more in wounded leaves [57,58].

Analyzing the mechanisms of the Ca^2+^-dependent regulation of JA biosynthesis, it can be noted that along with the increase in the JA and JA-Ile concentrations, there was an increase in the concentration of their precursor OPDA [30,51,57]; therefore, Ca^2+^ more likely affects the early steps of JA biosynthesis. This is consistent with the suggestion [10] that a Ca^2+^-dependent enzyme, which is involved in the early steps of the biosynthesis before OPDA (Box 2), participates in the rapid synthesis of JAs in systemic leaves upon local wounding. This enzyme is 13-lipoxygenase (LOX), which catalyzes the synthesis of JA precursors. For one of them, it is shown that LOX6 contributes to the rapid JA synthesis in systemic *Arabidopsis* leaves under the local wounding [27]. This appears to be a direct activation by Ca^2+^: LOX6 is predicted to have a β-barrel domain that may bind Ca^2+^ [10].

The initial steps of JA biosynthesis (Box 2) also involve phospholipases, whose activity may depend on [Ca^2+^]_cyt_. It is known that the increase in JA production after wounding can be mediated by changes in PLD activity: influx of Ca^2+^ induces the translocation of PLD to membrane, where it releases polyunsaturated fatty acids, which are necessary for the JA synthesis, from membrane phospholipids [59,60,61].

The role of the Ca^2+^ signaling system in the regulation of JA production is also evidenced by works on the involvement of Ca^2+^ sensors. The decoding of [Ca^2+^]_cyt_ elevations is mediated by various Ca^2+^ sensors, including calmodulins (CaMs), calmodulin-like proteins (CMLs), calmodulin-binding proteins (CBPs), calcineurin B-like proteins (CBLs), CBL-interacting protein kinases (CIPKs) and calcium-dependent protein kinases (CDPKs).

The wound-induced [Ca^2+^]_cyt_ elevation activates CaM, which in turn activates a protein kinase that phosphorylates JASMONATE-ASSOCIATED VQ-MOTIF 1 (JAV1). JAV1 is a part of the JAV1-JAZ8-WRKY51 repressor complex, which inhibits expression of JA biosynthesis genes. Phosphorylation of JAV1 causes dissociation of this complex, which removes transcriptional repression of JA biosynthesis genes and thus leads to production of JAs [62].

The Ca^2+^ sensor protein, CML37, functions as a positive regulator in Ca^2+^ signaling upon wounding by herbivore, connecting Ca^2+^ and JA signaling. The herbivore-induced JA-Ile and OPDA elevations decrease in *CML37* loss-of-function plants [42]. Another Ca^2+^ sensor protein, CML42, does not affect the herbivore-induced and the wound-induced JA, JA-Ile and OPDA elevations [25]. A study of JA content in rice roots showed that OsCBL1 is a positive regulator of JA biosynthesis, as the JA content in *OsCBL1*-knockdown plants was decreased compared to the wild-type plants [63].

CDPKs are also involved in the regulation of stress-induced JA levels, as evidenced by the activation of CDPK2 in tobacco plants (*Nicotiana benthamiana*), which causes the increase in the JA and OPDA content. Transient expression of constitutively activated *NtCDPK2* causes increased JA and OPDA production at rest, which increases under stress and exceeds the production in control plants [64]. Whereas CDPK2 positively regulates JA biosynthesis, CDPK4 and CDPK5 suppress JA biosynthesis [26,41]. It was found that simultaneously silencing *Nicotiana attenuata NaCDPK4* and *NaCDPK5* results in overaccumulation of JA and JA-Ile at rest and after wounding or simulated herbivory treatments [26]. The further investigation of the mechanisms by which NaCDPK4 and NaCDPK5 negatively control JA biosynthesis showed that OPDA concentration was also increased in mutants, but there were no differences in transcript levels of JA biosynthesis enzymes between mutants and the wild-type plants, suggesting that NaCDPK4 and NaCDPK5 have the role of regulators of the enzymatic activity in the early steps of JA biosynthesis [41].

Thus, the final target of Ca^2+^-dependent regulation of JA production may be both the expression of JA biosynthesis genes and the activity of JA biosynthesis enzymes, most likely in the early steps. The suggestion of Ca^2+^-dependent regulation of enzyme activity is consistent with the rapid stress-induced production of JAs, confirmed by much experimental data on different plant species and under various local stimuli (Section 2.1).

Another mechanism of JA biosynthesis induction may be pH changes, which, along with the Ca^2+^ wave, accompany ESs [4]. The shifts during the ES generation are most likely mediated by changes in the activity of H^+^-ATPase [4]. A recent study has shown that changes in H^+^-ATPase activity affect the content of JAs. The study was carried out in unstimulated leaves of *Arabidopsis* plants under local wounding and it showed that in mutant plants with reduced function of H^+^-ATPase (AHA1), there is the increased accumulation of JAs after wounding compared to the wild type [65]. Furthermore, loss of *AHA1* function affects pH at rest, causing less medium acidification compared to the wild type [65], suggesting a role of H^+^-ATPase AHA1 in pH regulation. Therefore, the increasing JA production in mutant plants was apparently associated with pH shifts.

Along with Ca^2+^ and H^+^, other ions involved in the generation and the propagation of ES can also be considered as potential mediators of hormone responses. Stimulus-induced changes in the content of JAs can also be affected by anion fluxes due to the involvement of anion channels in the ES generation [6,7,8,10,66]. At present, there is evidence of the involvement of anion stretch-activated MscS (mechanosensitive channel of small conductance)-like (MSL) 10 in the systemic VP propagation in response to wounding in *Arabidopsis* [67,68]. The mutation of *MSL10* reduces the duration of VP and the expression of JA pathway marker gene *JAZ10* in distal leaves [68]. Another study using *Arabidopsis* plants carrying a gain-of-function mutation in *MSL10* showed higher wound-induced JA and OPDA production in mutants than in wild-type plants [69]. Taken together, these findings suggest that anions could be potential mediators of ES-induced changes in concentration of JAs.

One of the integral elements of ES generation are K^+^ channels. However, whether K^+^ channels may be involved in stimulus-induced JA production is unclear. It is suggested that ARABIDOPSIS K^+^ TRANSPORTER 1 (AKT1) can play a role in the negative regulation of JA signaling by mediating K^+^ influx [70], but the role of AKT1 in ES is unclear to date. Other K^+^ channels, such as AKT2/3 and GUARD CELL OUTWARD RECTIFYING K^+^ (GORK), are involved in ES generation and propagation [7,10,71,72], and their roles in the control of JA production may need to be studied further.

Another potential mechanism of the regulation of JA biosynthesis may be changes in ROS levels. Evidence of the regulation of JA concentration by ROS can be seen in experimental data on the simulated induction of the increase in the content of JAs upon exogenous ROS treatment [52,73,74], as well as altered JA levels in mutant plants with loss of function of NADPH oxidase, a member of the respiratory burst oxidase homologs (RBOHs) family. Stimulus-induced increase in JA and OPDA concentrations in systemic *Arabidopsis thaliana* leaves was attenuated in *rbohD* mutants [32]. The stimulation of JA production in *Arabidopsis thaliana* [74] and *Panax ginseng* plants [73] required an increase in H_2_O_2_ concentration, dependent on the activity of NADPH oxidase. In *Aquilaria sinensis* plants, accumulation of JAs in response to wounding also required the increase in concentration of H_2_O_2_ [75]. *RBOH1*-dependent ROS production is essential for JA biosynthesis regulation in *Solanum lycopersicum* in response to nematode attack [52].

Mitogen-activated protein kinases (MAPKs) are other candidates for the role of JA biosynthesis regulators, which are rapidly activated in response to wounding in both local and systemic tissues, and it is assumed that this activation is mediated by ESs [76]. Two MAPKs in *Nicotiana tabacum*, WOUND-INDUCED PROTEIN KINASE (WIPK/MAPK3) and SA-INDUCED PROTEIN KINASE (SIPK/MAPK6), are activated in response to wounding, which subsequently leads to the increase in the JA synthesis [77,78,79]. The kinase activity of WIPK in local tissues increased 3 min after wounding, peaked at 5 min and declined to the basal level at 30 min. In systemic tissues, the WIPK activity also increased at 3 min, reached a maximum at 10 min time point and declined to the basal level at 30 min [78]. These dynamics of WIPK activity are consistent with the dynamics of JAs described in Section 2.1, which suggests the role of WIPK in the rapid induction of JA biosynthesis. In another work, silencing genes encoding WIPK and SIPK in *Nicotiana attenuata* inhibits JA and JA-Ile biosynthesis induced by simulated herbivory or mechanical wounding [15]. Another MAPK in *Nicotiana attenuata*, MPK4, acts as a negative regulator of JA synthesis upon simulated attack of *Manduca sexta*, but does not affect JA levels upon wounding or the simulated attack of *Spodoptera littoralis* [76], which may be the mechanism for the specificity of hormone dynamics, described in Section 2.1.1. In rice, herbivore attack activates MPK3, which positively regulates JA accumulation [80]. In *Solanum lycopersicum*, the induction of JA synthesis in response to nematode attack was mediated by MPK1/2, the activation of which depends on interdependent electrical and ROS signals [52]. The specific mechanism of regulation of JA biosynthesis by MAPKs is still unknown. It is assumed that SIPK regulates the lipase, which releases linolenic acid from the chloroplast membrane, and WIPK probably affects the activity of allene oxide synthase [22,76]. Taken together, these studies indicate that the ES-mediated activation of MAPKs is involved in the regulation of JA biosynthesis and is part of initial plant reaction to mechanical wounding and herbivore attack.

**Figure 3:**
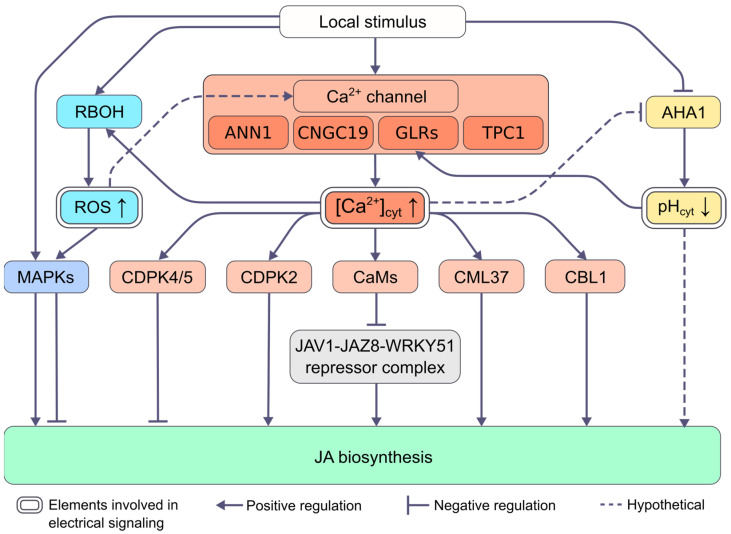
Model for regulation of stimulus-induced JA biosynthesis by electrical signals. Local stimulus causes the generation and propagation of electrical signals, which are accompanied by changes in pH, Ca^2+^ and ROS levels, mediated by changes in the activity of H^+^-ATPase AHA1, Ca^2+^ channels and NADPH oxidase RBOH, respectively. Altered pH, Ca^2+^ and ROS levels induce changes in the activity of downstream responders that eventually regulate JA biosynthesis. Abbreviations: AHA1, H^+^-ATPase 1; ANN1, ANNEXIN 1; [Ca^2+^]_cyt_, cytosolic Ca^2+^ concentration; CaMs, calmodulins; CBL1, calcineurin B-like protein 1; CDPK, calcium-dependent protein kinase; CML37, calmodulin-like protein 37; CNGC19, CYCLIC NUCLEOTIDE GATED CHANNEL 19; GLRs, GLUTAMATE RECEPTOR-LIKE proteins; JA, jasmonate; JAV1, JASMONATE-ASSOCIATED VQ-MOTIF 1; JAZ8, JASMONATE ZIM-domain protein 8; MAPKs, mitogen-activated protein kinases; pH_cyt_, cytosolic pH; RBOH, respiratory burst oxidase homolog; ROS, reactive oxygen species; TPC 1, TWO-PORE CHANNEL 1.

The regulation of JA biosynthesis also may be mediated by crosstalk of key signaling elements, involved in electrical activity. As discussed above, the induction of JA biosynthesis may be caused by pH shifts, which accompany the generation of ESs and depend on the H^+^-ATPase activity. In turn, the activity of H^+^-ATPase can be Ca^2+^-dependent [81,82] and regulated by CDPK [83], which is also confirmed by the associated transient changes in pH and Ca^2+^ concentration in cytosol in response to wounding [84]. On the other hand, pH shifts may affect [Ca^2+^]_cyt_: GLRs are regulated by pH [85]. Interdependence of MSL10 and GLRs in distal wound signaling [68] also supports the hypothesis of crosstalk-mediated regulation of JA biosynthesis, in this case mediated by the crosstalk of anion fluxes and Ca^2+^-waves. The Ca^2+^-dependent regulation of another key signaling element, RBOH, mediated the ROS wave, is also well known in the literature [2]. In turn, the activity of Ca^2+^ channels can be regulated by ROS [86,87]. ROS can also activate a MAPK phosphorylation cascade, which can form a positive amplification loop with ROS [88]. Suggested mechanisms of JA biosynthesis regulation by ESs described in this section are summarized in Figure 3.

Mechanisms of regulation of the stress-induced increase in ABA content by ESs are not sufficiently studied; nevertheless, several potential ways can be assumed. First of all, the involvement of Ca^2+^, ubiquitous signaling agents, can link ABA production and ESs, although it is known that loss of *CNGC19* function and the resulting attenuated stimulus-induced [Ca^2+^]_cyt_ elevation do not affect ABA content [51]. However, this role can be played by other Ca^2+^ channels that have not been identified to date. It can be suggested that there are mechanisms regulating ABA content through various Ca^2+^-binding proteins. It is shown for CBL9 that it can modulate ABA biosynthesis under abiotic stress. The disruption of *CBL9* gene function in *Arabidopsis thaliana* leads to increased accumulation of ABA under the stress conditions compared to the wild type. Furthermore, expression of *CBL9* was inducible by ABA, thereby representing a crosstalk node in connecting ABA signaling and ABA biosynthesis under stress conditions [89]. Another Ca^2+^-binding protein, CML42, also negatively affects ABA biosynthesis under drought, since it was observed that ABA levels were higher in *cml42* mutants than in wild-type plants [25]. Taken together, these data suggest that Ca^2+^ may play a negative role in the regulation of ABA production and that its function is mediated by various Ca^2+^-binding proteins.

The regulation of the biosynthetic gene expression through a Ca^2+^-dependent phosphorylation cascade, which is activated in response to abiotic stresses, is also suggested for ABA, and ABA feedback can stimulate the expression of the biosynthetic genes also through the Ca^2+^-dependent phosphorylation cascade [90]. On the whole, for many phytohormones, a positive feedback loop involving Ca^2+^ and ROS is assumed, and an increase in biosynthesis [88].

Taking into account the close relationship between Ca^2+^ and ROS [87], ROS can be assumed to be a regulator of ABA levels. It is shown in *Arabidopsis thaliana* that the rapid (10 min) transient increase in ABA content in systemic tissues in response to local heat stress was suppressed in *rbohD* mutants [34]. It also showed that both ROS wave and ESs were suppressed in *rbohD* mutants [34]. Taken together, this suggests that ESs and ROS waves could function cooperatively to control ABA production.

The ABA content in plants also can be potentially regulated by pH. It is known that dehydration stress causes an increase in apoplastic pH and ABA concentration, which were completely eliminated after pretreatment with fusicoccin, activating H^+^ extrusion and thereby contributing to apoplast acidification [91]. This increase in ABA concentration may be implemented by hydrolysis of ABA precursors in response to the increase in apoplast pH under stress conditions [92,93].

A potential point of controlling ABA content by pH may be the regulation of transport processes of ABA conjugates, whose deconjugation enzymes are separated by cellular compartments. ABA can be produced by deconjugation of the ABA glucosyl ester (ABA-GE), which is a storage or transport form of ABA and accumulated in the vacuole, apoplast and endoplasmic reticulum (ER) [94]. Deconjugation of ABA-GE by ER and vacuolar β-glucosidases allows a rapid formation of the free ABA in response to abiotic stress conditions such as dehydration and salt stress. Deconjugation of ABA-GE may be important in rapid stress reactions, as the free ABA is generated in a one-step hydrolysis reaction. This reaction occurs in vacuoles or ER; therefore, it requires the import of ABA-GE into these compartments. The import of ABA-GE into vacuoles is mediated by two distinct membrane transport mechanisms: proton-dependent antiport mechanism and ATP-binding cassette (ABC) transporters [95]. The effect of orthovanadate, ABC transporter inhibitor, on vacuolar ABA-GE import by AtABCC2 showed the decrease in ABA-GE uptake [95], which presumably may also affect the decrease in free ABA concentration.

The regulatory role of pH may be realized by changing the expression of ABA biosynthesis genes. It is shown that changes in intracellular pH are sufficient to modulate the expression of ABA biosynthesis genes and ABA accumulation [96]. Pretreatment of plants with chemical agents that change cytosolic pH affects ABA concentration both at rest and under osmotic stress. It is also noted in this work that ABA biosynthesis does not seem to linearly correlate with the absolute pH value in any compartments, but rather depends on the pH gradient between the cytoplasm and the vacuole (or another compartment) [96]. Thus, stress-induced ABA biosynthesis is more likely affected by disturbed intracellular pH homeostasis.

Anion and K^+^ fluxes during ES generation can probably also mediate stimulus-induced ABA production. ES generation in higher plants is accompanied by anion and K^+^ efflux with significant and prolonged changes in the intra- and extracellular concentrations of these ions [6], which can lead to changes in the turgor of plant cells. It was also demonstrated that ABA biosynthesis is triggered by a reduction in leaf turgor. Moreover, an increase in ABA levels was observed over a short timeframe following leaf exposure to high vapor pressure deficit, a method of modifying leaf cell turgor by the application of external pressure [97,98,99].

Mechanisms of regulation of SA biosynthesis by ESs can also be proposed. As in the case of ABA, loss of *CNGC19* function and the resulting attenuated stimulus-induced [Ca^2+^]_cyt_ elevation do not affect SA content [43,51]. Nevertheless, some Ca^2+^-dependent mechanisms of SA content regulation can be suggested, since Ca^2+^ influx into the cell may be mediated by other Ca^2+^ channels involved in long-distance signaling [2,7]. There are several Ca^2+^-dependent proteins involved in the regulation of SA biosynthesis. In *Arabidopsis*, *CDPK1* overexpression results in enhanced resistance to bacterial and fungal pathogens, which coincides with constitutively high levels of SA, and correspondingly increased expression of SA biosynthesis genes [100]. In transgenic *Arabidopsis* with *CDPK5* overexpression, enhanced resistance against bacterial infection is dependent on high SA levels, and plants display constitutive SA signaling [100,101]. In *Nicotiana attenuata,* CDPK4 and CDPK5 are not involved in the regulation of SA biosynthesis: simultaneously silencing *NaCDPK4* and *NaCDPK5* does not affect SA levels at rest or after damage, induced by wounding or herbivore [26]. Another Ca^2+^ sensor protein, CML42, does not affect SA levels at rest or under herbivore attack [25]. Positive regulation of SA biosynthesis by Ca^2+^ may be realized through CBP60g, whose activity is modulated by Ca^2+^/CaM at the post-transcriptional level [101].

Another potential regulator of SA biosynthesis may be the change in the activity of MAPKs. In tobacco plants, MAPKs WIPK and SIPK regulate SA levels upon wounding. In *WIPK/SIPK*-silenced plants, wounding induces increased SA accumulation compared to the wild type [77,79]. In another study, silencing genes encoding WIPK and SIPK in *Nicotiana attenuata* inhibits SA biosynthesis induced by simulated herbivory or mechanical wounding [15]. In rice plants, silencing *OsMPK3* does not affect basal and herbivore-induced SA levels [80]. Taken together, the studies described above show a complex and contradictory picture of possible regulation of SA production by ESs. Along with examples of positive regulation, there is a lot of evidence of the negative regulation of SA biosynthesis or no effect on SA content. It is possible that there is interplay between various hormones, which also affects the production of phytohormones.

Thus, mechanisms of regulation of changes in phytohormone content by ESs can potentially be mediated by changes in Ca^2+^, H^+^ and ROS concentrations, which accompany the generation and propagation of ESs. For some mechanisms, their involvement can be considered an established fact, but for most, confirmation is required.

## 3. The Role of Electrical Signals and Phytohormones in the Formation of a Systemic Response

A systemic response to various local stimuli requires coordinated transmission of signals throughout the plant, among which electrical and chemical signals play a significant role. It is well-known that the spectrum of functional responses induced by such signals necessary for plant survival and adaptation is extremely diverse (Box 3) and includes movements, changes in photosynthesis activity, changes in transpiration and respiration rates, induction of gene expression, etc. [6,8,102]. The time range in which systemic responses induced by long-distance signals occur is very wide—from several seconds, such as movement reactions when a *Dionaea muscipula* trap closes [103], to a day or more, as in the case of changes in the rate of growth and development [104]. In most cases, the formation of a systemic response to local stimuli occurs within minutes and hours, such as changes in the activity of photosynthesis, transpiration, the level of metabolites and digestive enzymes in carnivorous plants [32,34,105,106,107]. It should be noted that the temporal dynamics of the systemic response are quite complex; in some cases, the systemic response clearly distinguishes between short-term and long-term phases of the response, such as for changes in photosynthesis activity [35,39,108,109], stomatal conductance [35,110,111] and ATP content [112]. However, despite the fact that the mechanisms of systemic response formation and the contribution of signals of different nature to the induction of each of the response phases remain poorly understood, the main features of this process can be distinguished.

Box 3 **The electrical signal induces various responses, among which a number of the most significant can be noted.**

photosynthesistranspirationrespirationmovementsproduction of metabolitesphytohormone productionTransport processesreproductive processesgene expressiongrowth processesmorphogenesisetc.

Since the development of a systemic response begins almost immediately after the arrival of a long-distance signal to the unstimulated parts of the plant, among which, as noted above, ESs are among the most rapid, it can be assumed that these signals are responsible for the induction of the fast phase of the response [1,2,6]. It is known that the mechanisms of the induction of systemic response by ESs are based on changes in ionic concentrations that take place during their generation [6,8]. A special role belongs to Ca^2+^ and H^+^, the concentration of which increases in the cell both during AP and VP, and which are important mediators [1,2,6,8,9,10], capable of performing the so-called physiological regulation that directly affects physiological processes. It should also be noted that the VP propagation is associated with an increase in the levels of H_2_O_2_, which is a ubiquitous signaling agent [1,2,6,9,10].

The mechanisms of induction of a systemic response by an electrical signal can be analyzed using the example of the response of photosynthesis, as these responses are the most studied. The ES propagation causes a transient decrease in the activity of photosynthesis, manifested as a decrease in the rate of CO_2_ assimilation [105,109,113,114,115], and in changes in the parameters of the light reactions of photosynthesis—a decrease in the quantum yield of photosystem II, an increase in non-photochemical quenching (NPQ) [35,39,105,109,116]. The fast response phase, which usually proceeds no more than ten minutes, starts almost immediately after the occurrence of ES, and the lag period usually does not exceed several tens of seconds [108,113,114,116,117]. A considerable role in the rapid decrease in the activity of photosynthesis belongs to the pH changes occurring during the generation of ES [109,113,114,118], which lead to the inactivation of the Calvin–Benson cycle [5,119], the increase in NPQ [120] and reduced availability of CO_2_ for chloroplasts by changing the CO_2_:HCO_3_ ratio [118,121] or activity of aquaporins [113].

As mentioned earlier, the photosynthesis response includes two phases [35,39,108]. Changes in ionic concentrations during ES are responsible for the induction of the first short-term phase, and the second long-term phase is, apparently, regulated by hormones. Moreover, this effect is primarily due to a decrease in the availability of CO_2_ due to the closure of stomata [122]. Suppression of the rate of CO_2_ assimilation has been demonstrated with an increase in the level of JA [36,123,124] and ABA [36,123,125], but there are data on the effect of ABA on the light parameters of photosynthesis [122,125]. Note that the duration of changes in photosynthesis activity caused by hormones such as JAs can be up to several days [122,124]. As signaling molecules, hormones are able to directly regulate the activity of key physiological processes, both by changing the activity of enzymes, transporters, etc. [126,127], i.e., through physiological regulation, and by changing the level of gene expression.

The mechanisms of regulation of physiological processes by hormones are well studied in relation to the functioning of the stomatal apparatus. In general, the mechanism of stomatal closure is a decrease in the turgor of guard cells due to a decrease in osmotic pressure. This is mainly due to the efflux of K^+^, Cl^−^ and malate ions from the cell, in some cases with the participation of H^+^ and Ca^2+^ acting as inducers of these ion fluxes [128,129]. The mechanisms of action are best studied for ABA [32,37,111,123,125,130,131,132,133,134,135,136,137] and JAs, including JA [36,37,123,124,135,138,139,140,141], JA-Ile [142] and methyl jasmonate (MeJA) [133,137,138,143,144,145], including their precursor OPDA [136,142]. The process of JA- [133] and ABA-induced [32,125,132,133,146] stomatal closure is largely similar and includes activation of NADPH oxidase, presumably RBOHD and RBOHF [32,132], which produces ROS. ROS, in turn, activate the Ca^2+^ channels of guard cells [133,134,146]. The increase in [Ca^2+^]_cyt_ induced by channel opening leads to the activation of S-type anion channels [133,147], presumably SLOW ANION CHANNEL-ASSOCIATED 1 (SLAC1) in the case of ABA [32,147,148], and K^+^ channels (presumably GUARD CELL OUTWARD RECTIFYING K^+^ channel (GORK)) in the case of JA [140] and MeJA [137,145], which directly lead to the closure of the stomata. Of note, taking into account the available data, there is a direct crossing of the mechanisms or interaction between JA and ABA at the level between the production of ROS and the activation of Ca^2+^ channels [133,140,143,149].

However, it should be noted that the presented mechanism is not the only one. There are a number of works suggesting NO produced by H_2_O_2_-activated nitrate reductases as a key activator of Ca^2+^ channels causing ABA-dependent stomatal closure [130,133,150]. Moreover, an alternative, Ca^2+^-independent mechanism of stomatal closure has been suggested for ABA [137,151].

In the case of SA, which is also shown to be involved in stomatal closure [111,152,153,154], the induction mechanism is somewhat different—the main role is played by salicylhydroxamic acid (SHAM)-sensitive peroxidases [110,111,153], which produce ROS (H_2_O_2_ and O^2−^) in response to SA, whereas NADPH oxidases are not involved in this process [32]. Probably, this process also involves ROS- [110] or Ca^2+^-dependent production of NO [155]. Compared to the mechanisms induced by JAs and ABA, in SA-induced stomatal closure, Ca^2+^ channels are not directly involved, and stomatal closure occurs through the suppression of extracellular Ca^2+^ binding and the suppression of the activity of K^+^ channels [110,154]. Despite the noted differences in the mechanisms of stomatal closure with the participation of various hormones, one can distinguish universal features of the process, which include the determining role of ROS [32,110,150] and changes in ionic concentrations, primarily of Ca^2+^ [133,134].

Along with inducing a response by altering the activity of enzymes and transporters, hormones can induce long-term changes by regulating gene expression. Continuing to consider the formation of a systemic response using the example of photosynthesis, one can note that a significant number of genes associated with it are controlled, in particular, by JAs [156]. Thus, it has been shown that MeJA suppresses the expression of genes responsible for the synthesis of chlorophyll [122,156,157], the functioning of ribulose-1,5-bisphosphate carboxylase/oxygenase (RUBISCO) [122,156] and the formation of photosystems with light harvesting complexes [122,157] and electron transport systems [124]. The noted processes can lead to long-term suppression of photosynthesis activity in unstimulated parts of the plant. Thus, it can be concluded that the long-term phase of the response may be associated with a change in the content of hormones. It should be noted that gene expression leading to long-term changes in the physiological state can also be triggered directly by ESs, presumably through changes in [Ca^2+^]_cyt_ [107,158,159] or inositol-1,4,5-trisphosphate [107].

Considering the systemic response induced by hormones, it is necessary to note the specificity of the changes caused by them. In particular, this is due to the specific profile of gene expression in relation to certain hormones. Thus, JAs are well known as inducers of a defensive response, activating defense genes specific for attacks by various herbivores or pathogens [122,160,161], which is confirmed by the inhibition of growth of insects and pathogens that feed on plants with increased JA production [162]. Without delving into this topic, which is well covered in an excellent review by Erb and Reymond [163], we note that in the case of an insect attack, JA-dependent expression of a number of genes was also shown, including regulators of the production of JAs themselves, such as CORONATINE-INSENSITIVE 1 (COI1), JAZ, bHLH (basic helix–loop–helix), transcription factor MYC2 (also known as JIN1) [160,161,163,164], LOX2 [165] and protective genes such as hevein-like protein (HEL) [165]. Moreover, expression of several of those begins already within 5 min, closely correlating with an increase in the levels of JA and JA-Ile [164]. In addition to defense against herbivores and pathogens, JA-induced expression is observed for a number of abiotic stimuli, such as high light [33,34], burning [14] and temperature increase [33,166] or decrease [167,168]. In carnivorous plants, JAs are involved in the production of digestive enzymes in response to prey capture by the tentacles [106].

The SA regulates the expression of various protective genes, primarily those responsible for the redox balance [169,170,171], which occurs not only upon pathogen attacks, but also probably during local heat and light stimuli [33,170,172].

The ABA is characterized primarily by an increase in the expression of genes responsible for drought resistance [94,126]; however, drought cannot be attributed to local stressors, so we do not review them in this work. ABA-induced systemic responses are controlled, among other things, by the expression of genes of such important elements of signaling cascades as NADPH oxidase [132] and ascorbate peroxidase 2 (APX2), probably through the mediation of ROS [173], and expression of other genes under local stimuli such as high light [34,125,173].

It is important to mention the interaction of hormones for the induction of the expression of a number of genes, such as ABA-dependent induction of proteinase inhibitor 2 (PIN2) gene expression during wounding, which includes the involvement of JA [135]. It should be noted that the activation of many JA-inducible genes upon stress requires ABA production upstream of JA [143,168]. SA can act antagonistically towards JA in the regulation of gene expression [174,175]. The interaction of hormones also includes the fact that OPDA, apparently, also has a separate, ABA-independent mechanism of regulating stomatal closure [136].

This brief review of the regulation of gene expression by various hormones allows us to conclude that the gene expression profile is specific to various stimuli, which allows one to induce a stimulus-specific long-term systemic response. This is provided both by the stimulus-specific dynamics of hormones, discussed above, and by the interaction of hormones with each other. However, the mechanisms that provide specific changes in the content of hormones remain unknown. There is no doubt that ESs can induce an increase in concentrations of hormones [10,39,48]. Nevertheless, a specific profile of hormone dynamics is formed, apparently not only due to ES propagation, but also of signals of a different nature. It can be assumed that such signals are mRNA and other elicitors of herbivores and pathogens [163]. These facts suggest a possible multicomponent and multilevel regulation of specific changes in hormone concentrations under various stimuli, as well as the specific response caused by them.

**Figure 4:**
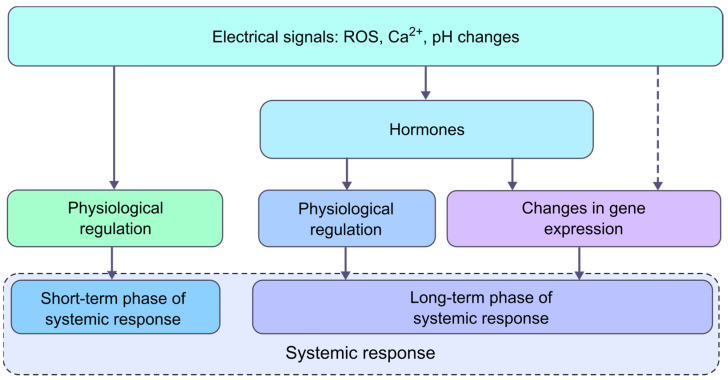
Model for the formation of two-phase systemic response. The propagation of electrical signals induces a short-term phase of the systemic response and changes in hormone content in the systemic tissues of plants. The short-term phase of the systemic response is controlled by so-called physiological regulation, which directly affects physiological processes mediated by changes in pH, Ca^2+^ and ROS levels. The long-term phase of the systemic response is controlled by hormones through physiological regulation and changes in gene expression, which can also be affected by electrical signals.

In summary, the analysis of the systemic response induced by ESs and hormones to local stimuli showed that in many cases, they cannot be considered as independent. As we mentioned above, many systemic responses induced by ESs, such as gene expression, transpiration, etc., occur in most cases through the mediation of hormones, which gives reason to consider them as links of the chain of the formation of a systemic response (Figure 4) [35,50,106]. At the same time, hormones also interact with other signaling systems and messengers, such as Ca^2+^ and ROS [7,12,88]. This becomes obvious due to the changes in the concentration of some phytohormones, mainly JA, ABA and SA, shown in a number of works in unstimulated parts of the plant associated with ESs, i.e., it is ESs that induce changes in the levels of these hormones [37,48,65]. Additionally, this is also supported by the dependence of changes in hormone content on VP parameters, shown in a number of studies [35,37,39]. It should also be noted that the development of a systemic response includes the same set of second messengers, namely ROS and Ca^2+^. Taking into account that the systemic response includes short- and long-term response phases, it turns out that a fast response phase is regulated by ESs, primarily due to changes in the concentrations of Ca^2+^, H^+^ and ROS, which simultaneously trigger hormone production. An increase in hormone concentrations leads to the formation of subsequent long-term phase of a systemic response due to changes in gene expression, etc. The regulation of the response to the action of stressors is carried out by the interaction of ESs with hormones and by the interaction of hormones with each other. The coordinated contribution of the hormonal and electrical systems ensures the formation of a systemic response in accordance with information about the stimulus.

## 4. Conclusions

Plants have a complex system of perception and transmission of information about external environmental stimuli, which involves several different signaling systems, among which hormonal and electrical systems play an important role in the formation of the systemic response in the entire plant’s organism. The integration of ESs and phytohormones allows plants to effectively coordinate their physiology and development. ESs are essential for rapid long-distance transmission, whereas ES-induced changes in phytohormone content mediate more specific physiological responses. However, there are still a number of outstanding questions. Despite major advances in defining molecular mechanisms involved in the generation and propagation of distance signals, the nature of these signals has not been fully elucidated. Specific ion channels and pumps, as well as enzymes, have only just begun to be identified, and the mechanisms of their interaction with each other are even less understood.

To date, significant progress has been made in understanding the propagation speed of ESs, Ca^2+^ and ROS waves and the timeframes of changes in phytohormone levels, providing information about the hierarchy and potential ways of integrating electrical and hormonal signaling systems. However, molecular mechanisms, which translate stimulus-induced changes in electrical activity into changes in the content of phytohormones, still remain largely unknown and require further investigation.

To build a complete picture of the mechanisms of the interaction between electrical and hormonal signaling systems, it is necessary to study the effect of phytohormones on membrane potential changes, which is especially important for repeated stimuli, as the repeated stimulus acts under conditions of changed hormonal levels; therefore, the mechanisms of generation and propagation of the ES can be different from those at rest. The initial content of phytohormones in plants can also affect the parameters of the ES, causing various systemic responses. Regarding the latter, long-term measurements are required to study long-term responses for several days or more to establish the timeframes for systemic responses, since now, such studies are mainly limited to a duration of several hours or 1–2 days. Solving these and many other questions related to the interplay and integration of hormonal and electrical signals will be important for understanding the response to stress in plants in general and will contribute to the development of methods for managing crop resistance.

## Data Availability

The authors declared that data were contained in this manuscript.
